# Should we adjust health expenditure for age structure on health systems efficiency? A worldwide analysis

**DOI:** 10.1186/s13561-023-00421-2

**Published:** 2023-02-13

**Authors:** João Vasco Santos, Filipa Santos Martins, Joana Pestana, Júlio Souza, Alberto Freitas, Jonathan Cylus

**Affiliations:** 1 MEDCIDS – Department of Community Medicine, Information and Health Decision Sciences, Faculty of Medicine, University of Porto, Porto, Portugal; 2 CINTESIS - Centre for Health Technology and Services Research, Porto, Portugal; 3 Public Health Unit, ARS Norte, Espinho/Gaia, Portugal; 4 Centro Hospitalar de São João, Porto, Portugal; 5 Nova School of Business and Economics, Universidade Nova de Lisboa, Carcavelos, Portugal; 6 Department of Health Policy, London School of Economics and Political Science, London, UK; 7 European Observatory On Health Systems and Policies, London, UK

**Keywords:** Health system, Efficiency, Age adjustment, Frontier models

## Abstract

**Introduction:**

Healthcare expenditure, a common input used in health systems efficiency analyses is affected by population age structure. However, while age structure is usually considered to adjust health system outputs, health expenditure and other inputs are seldom adjusted. We propose methods for adjusting Health Expenditure per Capita (HEpC) for population age structure on health system efficiency analyses and assess the goodness-of-fit, correlation, reliability and disagreement of different approaches.

**Methods:**

We performed a worldwide (188 countries) cross-sectional study of efficiency in 2015, using a stochastic frontier analysis. As single outputs, healthy life expectancy (HALE) at birth and at 65 years-old were considered in different models. We developed five models using as inputs: (1) HEpC (unadjusted); (2) age-adjusted HEpC; (3) HEpC and the proportion of 0–14, 15–64 and 65 + years-old; (4) HEpC and 5-year age-groups; and (5) HEpC ageing index. Akaike and Bayesian information criteria, Spearman’s rank correlation, intraclass correlation coefficient and information-based measure of disagreement were computed.

**Results:**

Models 1 and 2 showed the highest correlation (0.981 and 0.986 for HALE at birth and HALE at 65 years-old, respectively) and reliability (0.986 and 0.988) and the lowest disagreement (0.011 and 0.014). Model 2, with age-adjusted HEpC, presented the lowest information criteria values.

**Conclusions:**

Despite different models showing good correlation and reliability and low disagreement, there was important variability when age structure is considered that cannot be disregarded. The age-adjusted HE model provided the best goodness-of-fit and was the closest option to the current standard.

**Supplementary Information:**

The online version contains supplementary material available at 10.1186/s13561-023-00421-2.

## Key-points

- Adjusting health expenditure per capita for population age structure is needed to avoid bias in health system efficiency analysis.
- Age-adjusted health expenditure per capita, through age standardization, is a good option for such adjustment.
- Similar approaches can be applied to other analyses or contexts using age adjustment.

## Introduction

Health system efficiency can be defined as the “ratio of resources consumed (health system inputs) to some measure of the valued health system outputs that they create” [1]. Efficiency is one of the major dimensions of health systems performance assessment [1, 2], and health systems’ efficiency is a priority due to finite resources and increasing demand for health care [1, 3, 4]. Comparing national health systems efficiency (rather than health care sectors only, such as hospital or primary care) is a field of long-standing interest, one which can help policy-makers to identify good performers, optimize the allocation of resources, and correct flaws that interfere with efficiency [5, 6].

Identifying the appropriate inputs and outputs when measuring the efficiency of health systems is not straightforward. Hollingsworth (2012) suggests that a set of guidelines is needed for efficiency analysis in health care to make them relevant for policy-makers, considering that there are no accepted methods and the diversity of variables and methods used make it difficult to compare results and apply them in other settings [7]. Given the absence of consensus, the current methodological choices must consider the objective of the analysis and the type and quality of the data used [8]. While health system outputs are often population health measures, health system inputs are often measured in the form of expenditures [1, 9]. It is also essential to decide on the environmental variables to include, i.e. factors that influence a health care system’s performance, which reflect the environment in which it operates and which are beyond the health system’s control [1, 9].

Health expenditure itself can depend on so-called environmental determinants such as population demographics or economic factors [10, 11]. The relationship between aging and health expenditure has been extensively studied, as aging is associated with changes in the type of healthcare needs and frequency of provision [12–15]. Some researchers find that the relationship between age and health spending is attributable to proximity to death and not calendar age itself [16]. Others argue that the direct link to expenditure might be through increased multi-morbidity at older ages [16–18]. Alternatively, other studies consider that the needs of an older population alone have a modest effect on increasing healthcare expenditure, with overall growth in health spending mainly attributed to growth in health prices and technological innovations [19–21].

Health system efficiency is likely to vary depending on the demographic structure of the population served, such as age and sex distribution [9]. Processes for health output standardization to account for the demographic structure have already been developed and can be summarized into three ways: restricting comparison to entities operating within similarly constrained environments; incorporating environmental factors using statistical methods, namely regression models; and adjusting outputs for external constraints using risk adjustment techniques [1]. However, the appropriate inclusion of age distribution on the inputs side is less clear and efficiency analyses, when accounting for the effects of age, use different approaches, such as splitting the population at 65 years-old or in age groups with different compositions [22–24]. This methodological variability is due to the lack of consensus on the method to be adopted, as well as to the lack of knowledge of the implications for efficiency analysis of using different approaches to adjust health expenditures for age.

Thus, we aimed to assess the impact of adjusting health expenditure for population age structure on health system efficiency scores, by evaluating goodness-of-fit, correlation, reliability, and (dis)agreement of different approaches. In this study, we also propose a method to adjust health expenditure for age, through a standardisation process.

## Methods

In this study, we performed a worldwide (188 countries) cross-sectional study of efficiency in 2015. Most studies on cross-country comparison of health system efficiency are conducted using cross-sectional data or repeated cross-sectional analysis using panel data instead [25]. A cross-sectional design has been chosen over a panel approach as we mainly intend to investigate and prove assumptions on distinct specifications, which in turn is considerably easy to conduct with cross-sectional data, apart from enabling the use of multiple variables that can only be available at a single or different points in time.

Considering that the different models used for age-adjustment aim to measure the same concept but differ in the definition and assessment of age structure, it is important to understand whether they lead to differences in the interpretation of results and how these differences occur across countries. In the first stage, we estimated the age-adjusted health expenditure per capita (HEpC) and compared it to the original HEpC, by assessing correlation, reliability, and (dis)agreement of these measures. In the second stage, we performed a stochastic frontier analysis (SFA) to estimate the countries’ efficiency scores using the different models resulting from age-adjustment measures. In the third stage, we evaluated goodness-of-fit, correlation, reliability, and (dis)agreement of efficiency scores across the different model specifications.

### Output variables

Population health indicators allow the assessment of the impact of public health policies and financial investments in health care services, despite limitations on population health measurement methods and difficulty in the association of health changes/impacts to specific policies or investments. In this study, each efficiency model included healthy life expectancy (HALE) at birth or HALE at 65 years old, reusing the estimates from the Global Burden of Disease (GBD) 2019 study of the Institute of Health Metrics and Evaluation (IHME) [26]. Health expectancies are population health metrics that combine both mortality and morbidity. In fact, HALE is calculated by life tables and using a combination of epidemiological data and disability weights for health states [27]. It can be defined as the average number of years that a person at a certain age would live in full health, i.e., in absence of disease or disability [28]. Life expectancy and health-adjusted life expectancy are the most commonly used outputs in system efficiency studies [9, 25] since they are a ‘reasonable’ objective for the institutional framework for which the analysis is undertaken [29]. However, the main goal of health systems is not merely restricted to an increase in life expectancy, but also to an improvement in quality of life by tackling a range of health issues that do not necessarily result in death. The choice of HALE is due to its capacity to account for the broader objectives of health systems when compared to life expectancy [30].

### Input variables

Total health expenditure per capita [in US$, adjusted for purchasing-power parity (PPP)] and age structure were the only inputs considered for the construction of the models.While health expenditure data was obtained via the Global Health Data Exchange (IHME), demographic data was retrieved from the World Population Prospects of the United Nations [31, 32]. The latter was included in different formats in order to evaluate the correlation, reliability, and (dis)agreement between different adjustments. Additionally, for robustness check, we have also used education attainment, i.e. age-standardized education per capita, as a control for the models, achieving similar results (education data was missing for 3 out of the 188 countries) as presented in the Supplementary tables [33].

Using the average HEpC of a subset of OECD countries (i.e. Australia, Canada, Germany, Japan, Netherlands, Switzerland and the United Kingdom) and adjusting all 188 countries’ age structure to the subset weighted (single year) age structure, we estimated an ageing index for each country, as described below [34]. Due to the lack of global data on HepC by age group, we used the OECD subset, which is a limitation of this study. In fact, this method (explained in detail below), is analogous to the diagnosis-related groups methodology for hospital financing, including relative weights’ calculation based on the average hospital episode cost of a country (or global average health expenditures per capita, in this study) and a case-mix index by hospital (in this case, by country).

The relative weights of each age group *g* were calculated through the ratio between the HepC of each age group and the HepC of the overall population. Therefore, for age group *g*, the following formula was applied:

$$\mathrm{Age}\;\mathrm{group}\;{\mathrm{weight}}_g=\frac{HEpC{}_g}{HEpC}$$

For example, for the 35–39 years-old age group, the calculated weight _*g*_ was 0.627, which suggests that the HepC of this age group is lower than the overall population HepC.

For the next step, in order to calculate the ageing index for each country *I*, we multiply the relative size of the age groups in the country *i* with its relative weight, as follows:

$$Ageing\;index_i=\frac{\sum_{g=1}^G(n_{ig}\times\mathrm{Age}\;\mathrm{group}\;{\mathrm{weight}}_g)}{n_i}$$

### Stochastic frontier analysis and models

Stochastic Frontier Analysis (SFA) is a parametric method, which was simultaneously developed by Aigner, Lovell and Schmidt, and by Meeusen and van den Broeck in 1977 [35, 36]. Using this approach we estimate several health production functions while modelling the inefficiency term, i.e. distance to the frontier, as a linear function of a set of explanatory variables.

A parametric approach was chosen over a nonparametric one due to its capacity to decompose the error term ($${e}_{i})$$ into the statistical noise term ($${v}_{i})$$ and the inefficiency term $$({u}_{i})$$, such as: $${e}_{i}={u}_{i}+{v}_{i}$$. Additionally, it is also useful for quantitatively and independently measuring and controlling for the effect of exogenous factors, which allows hypothesis testing with goodness of fit of the estimated models. Furthermore, nonparametric approaches such as data envelopment analysis (DEA) tend to be sensitive to outliers and measurement errors, as well as considering as full efficient those decision making units without peers [25]. In particular, the main reason determining the choice of SFA was due to the fact that it is less restrictive to variable constraints than DEA, providing greater flexibility to include the age variable in different formats.

We estimated a stochastic frontier health production function following the framework proposed by Battese and Coelli in a one-step approach in which coefficients of explanatory variables and efficiency scores are estimated jointly using the predictors proposed by Battese and Coelli [37, 38]. Thus, we depart from the function of a production

$${y}_{i}=f\left({x}_{i}, \beta \right)T{E}_{i}$$

with a single output ${y}_{i}$ that measures the health outcome, HALE in this case, in country *i*, a vector of inputs ${x}_{i}$ that represents the heath care production inputs associated with country *i*, β as the vector of parameters if the function to be estimated, and $T{E}_{i}$ as the technical efficiency for country *i*. The econometric model can be depicted linearly using the logs of the variables as follows:

$$\mathrm{ln}{y}_{i}={x}_{i}^{^{\prime}}\beta +{(v}_{i}-{u}_{i})$$

where ${(v}_{i}-{u}_{i})$ is randomly distributed across countries. We assume that ${v}_{i}$ is independent and identically distributed with a mean of zero and variance σ_v_^2^, and ${u}_{i}$ is a non-negative random component measuring technical inefficiency, assumed to be independently distributed such that ${u}_{i}$ is obtained through the half-normal distribution. Besides the half-normal distribution, other distributions are also described in the literature, such as exponential and truncated. In fact, these three are the most widely used due to better interpretation and ease of estimation, while there is no consensus on the best distribution option.

The technical efficiency (TE) scores of production of the *i*th unit are defined by the following equation,with the score ranging between 0 (very inefficient) and 1 (very efficient),:

$${TE}_{i}=\frac{{y}_{i}}{{e}^{\left({{x}^{\mathrm{^{\prime}}}}_{i}\beta +{v}_{i}\right)}}=\frac{{e}^{\left({{x}^{\mathrm{^{\prime}}}}_{i}\beta +{v}_{i}-{u}_{i}\right)}}{{e}^{\left({{x}^{\mathrm{^{\prime}}}}_{i}\beta +{v}_{i}\right)}}{=e}^{-{u}_{i}}$$

A total of five models per output were considered, differing only on the age adjustment and, thus, differing only in the vector of inputs in model ${x}_{i}$:

- Model 1 – health expenditure per capita only (without age);

The vector of inputs ${x}_{i}$ in this model only includes the health expenditure per capita (HEpC):

$$x_i=HEpC_i$$

- Model 2 – age-adjusted health expenditure per capita;

The age-adjusted health expenditure per capita for country *i* was obtained through the ratio between the country’s health expenditure per capita and the ageing index as follows:

$$x_i=\frac{HEpC_i}{Ageing\;index_i}$$

- Model 3 – health expenditure per capita + proportion population 0-14 years-old + proportion population 15-64 years-old + proportion population 65+ years-old;

$$x_i=HEpC_i+\frac{n_{ig_{0-14}}}{n_i}+\frac{n_{ig_{15-64}}}{n_i}+\frac{n_{ig_{65+}}}{n_i}$$

where ${n}_{ig}$ indicates the number of people in age group g in the country i, and ${n}_{i}$ the population in country I in all age groups.

- Model 4 – health expenditure per capita with population-adjusted metrics: proportions 5-year age-groups;

$$x_i=HEpC_i+\sum\nolimits_{g=1}^G\frac{n_{ig}}{n_i}$$

where $G$ is a vector of age groups from (1) age 0 to 4, (2) age 5 to 9, until (18) age 85 and over.

- Model 5 – health expenditure per capita + ageing index.

$$x_i=HEpC_i+Ageing\;index_i$$

### Goodness-of-fit, correlation, reliability, and (dis)agreement analysis

Following the Guidelines for Reporting Reliability and agreement studies [39], we evaluated correlation, reliability, and (dis)agreement between: (1) health expenditure per capita and age-adjusted health expenditure per capita; and (2) the efficiency scores of different models for each output variable. Two scores can be highly correlated though with poor agreement or reliability between them. Furthermore, while reliability can be defined as the ability of a measurement to differentiate between subjects/objects, agreement is the degree to which scores/ratings are identical.

To assess the correlation, Spearman’s rank-order correlation coefficient) of efficiency scores was calculated, between models, for each output variable. For this analysis, 10,000 bootstrap samples were drawn to calculate 95% confidence intervals (CIs). In this method, repeated simulations of the data generation process were performed, creating equisized new datasets. The original estimator obtained from each model is applied to each simulated dataset and the new estimates are produced by imitating the sampling distribution of the original estimator. Bootstrapping has gained popularity in efficiency analysis as it allows to derive statistical properties from efficiency scores, allowing to check and compare bias, variance and CIs of the assessed models [40].

Reliability was calculated through the intraclass correlation coefficient (ICC) (two-way random-effects model), which ranges from 0 (no reliability) to 1 (perfect reliability).

The information-based measure of disagreement (IBMD) was also calculated to evaluate (dis)agreement. IBMD is based on the amount of information contained in the differences among observations, ranging from 0 (no disagreement) to 1 (perfect disagreement).

Bland and Altman plots with limits of agreement were plotted, alongside 95% limits of agreement.

We also estimated the goodness-of-fit for each model, by calculating both the Akaike information criteria (AIC) and Bayesian information criteria (BIC) [41]. In order to validate the models and complement the goodness-of-fit, we have also conducted Likelihood ratio tests for SFA, as suggested by Coelli, in which we compared the fitted model with a corresponding model without inefficiency estimated by OLS [42].

We used R Studio version 2022.12.0 + 353 using R version 4.2.2 for data processing and statistical analysis.

## Results

In 2015, the 188 countries presented a median value of 64.0 (p25 57.1; p75 67.1) for HALE at birth and 11.8 (10.5; 13.7) years for HALE at 65 years-old, respectively. When adjusted for age, the median HE per capita increased from 814.0 US$ PPP (211.8; 1781.0) to 1100.0 US$ PPP (368.1; 2131.3). Table 1 displays the summary measures for non-adjusted and age-adjusted HE per capita, HALE at birth, and HALE at 65 years-old.

**Table 1:** Summary descriptive measures for healthy life expectancy (HALE) at birth and at 65 years-old, health expenditure (HE) per capita, age-adjusted HE per capita, ageing index, proportion of population (0–14 years old and 65 + years old) and efficiency scores by model, in 2015 for 188 countries

	Min	**p25**	Median	**p75**	Max	**Mean**	SD
HALE at birth (years)	42.4	**57.1**	64.0	**67.1**	73.9	**62.6**	6.4
HALE at 65 years-old (years)	7.6	**10.5**	11.8	**13.7**	16.9	**12.1**	2.1
HE per capita (US$ PPP)	12.0	**211.8**	814.0	**1781.0**	9780.0	**1406.2**	1699.9
Age-adjusted HE per capita (US$ PPP)	22.4	**368.1**	1100.0	**2131.3**	10,739.8	**1684.9**	1810.8
Ageing index	0.51	**0.59**	0.67	**0.87**	1.21	**0.73**	0.17
Proportion of population 0–14 years-old (%)	12.6	**18.3**	27.8	**38.5**	49.1	**28.5**	10.8
Proportion of population 65 + years-old (%)	0.7	**3.3**	5.7	**13.1**	27.3	**8.2**	6.0

*Min* minimum, *Max* maximum, *SD* standard deviation

Table 1 also displays the summary measures for the ageing index and the age proportion of the population (0–14 years-old and 65 + years-old). Considering all countries included, the ageing index presented a median value of 0.51 (p25 0.59; p75 0.87). The country with the highest proportion of individuals aged between 0 and 14 years-old was Niger accounting 49.1%, while the country with the highest proportion of individuals aged over 65 years-old was Japan accounting 27.3%.

Table 2 displays the summary measures for the efficiency scores for HALE at birth and at 65 years-old, by model. The median efficiency score was above 0.9 for all tested models, indicating that inefficiency is highly important to explain deviations from the production function, regardless of adjustments of health expenditure per capita for the age structure. Considering the maximum and minimum values calculated, these could only improve outputs (in this study, both measured as an increase in the healthy life expectancy at birth and at 65 years-old) by 1 to 32% across our analyses. Regarding models’ goodness-of-fit, AIC, BIC and the *p*-value of the LR test were the lowest for model 2, considering both outputs (i.e. HALE at birth and HALE at 65 years-old). Similar results were found using education attainment as control (Supplementary Table 1).

**Table 2:** Summary descriptive measures of efficiency scores and goodness-of-fit (Akaike and Bayesian information criteria and Likelihood ratio test) by stochastic frontier model of health system efficiency using healthy life expectancy (HALE) at birth and at 65 years-old as outputs, in 2015 for 188 countries

	HALE at birth										HALE at 65 years-old									
	Min	p25	Median	p75	Max	Mean	SD	AIC	BIC	LR P-value	Min	p25	Median	p75	Max	Mean	SD	AIC	BIC	LR P-value
Model 1	0.693	0.919	0.947	0.963	0.992	0.934	0.047	-511.6	-498.7	2.0 e-8	0.685	0.866	0.909	0.936	0.976	0.891	0.064	-279.7	-266.7	2.6 e-4
Model 2	0.684	0.910	0.946	0.963	0.993	0.929	0.051	-488.1	-475.2	0.9 e-8	0.666	0.861	0.903	0.930	0.975	0.885	0.068	-261.8	-248.9	1.1 e-4
Model 3	0.702	0.930	0.953	0.970	0.992	0.945	0.039	-574.5	-551.8	2.8 e-8	0.714	0.880	0.920	0.941	0.974	0.906	0.051	-305.8	-283.1	117.4 e-4
Model 4	0.710	0.936	0.957	0.973	0.994	0.948	0.037	-574.2	-503.0	18.1 e-8	0.686	0.873	0.918	0.949	0.981	0.905	0.058	-317.5	-246.3	12.3 e-4
Model 5	0.708	0.928	0.948	0.966	0.988	0.939	0.041	-539.3	-523.1	63.7 e-8	0.728	0.882	0.918	0.941	0.975	0.906	0.050	-297.8	-281.7	222.1 e-4

*Min* minimum, *Max* Maximum, *SD* standard deviation, *AIC* Akaike information criteria, *BIC* Bayesian information criteria, *LR* Likelihood Ratio

**Fig. 1:**
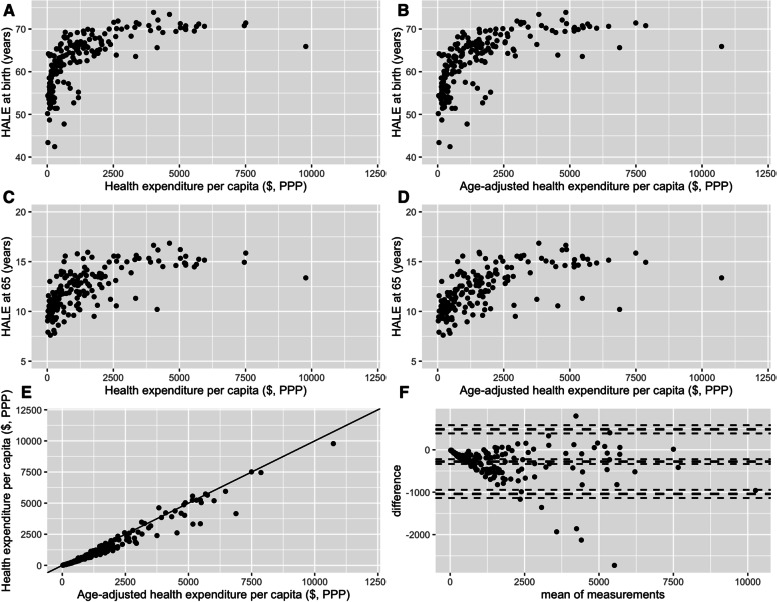
Estimated efficiency scores for HALE at birth (**A** and **B**) and HALE at 65 years-old (**C **and **D**), considering non-adjusted and age-adjusted HE per capita, respectively, in 2015 for 188 countries. Relationship between non-adjusted and age-adjusted HE per capita values € (**E**) and Bland–Altman plot for assessing the differences between all measurements (**F**). Horizontal lines show the mean difference and the 95% CI of limits of agreement

Figure 1 shows the plots of estimated efficiency values for HALE at birth and HALE at 65 years-old when comparing to non-adjusted and age-adjusted HE per capita. Each point within the plot represents a specific country. According to the visual analysis of the pair of graphs for HALE at birth and HALE at 65 years-old, it is possible to acknowledge a very similar pattern of results (A vs. B; C vs. D). The outlier value (above 10,000 $ PPP), which is common to the four graphs, corresponds to the United States of America.

Furthermore, the lower left graph of Fig. 1 (panel E) shows the relationship between non-adjusted and age-adjusted HE per capita values. Figure 1 (panel F) also presents a Bland–Altman plot (lower right) with 95% confidence interval limits for the differences between non-adjusted and age-adjusted HE per capita. The 95% limits were calculated assuming that the mean and standard deviation of the expenditures’ differences do not depend on the magnitude. This plot indicates a greater dispersion with the increase in the mean of HE per capita. Also, while the expected vast majority of estimated values lay within the confidence interval obtained from bootstrapping, the outlier cluster corresponds to the Persian Gulf countries, with the most extreme negative difference corresponding to Qatar.

**Fig. 2:**
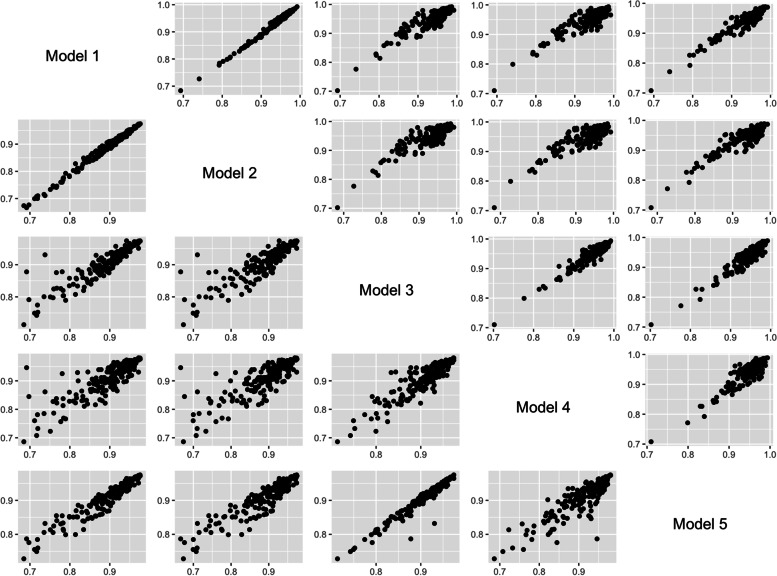
Scatterplot matrix of the pairwise efficiency estimates for HALE at birth (upper-diagonal) and HALE at 65 years (lower-diagonal), in 2015 for 188 countries

Figure 2 displays a scatter plot matrix for the efficiency scores from the models measuring efficiency in producing HALE at birth, in the upper right corner, and for HALE at 65 years-old, in the lower left corner, representing the relationship between the five models. Both figures show a positive relationship between the different models.

A good positive correlation was found among the five models. The highest values occur between models 1 and 2, both for HALE at birth (0.981) and HALE at 65 years-old (0.986) – Table 3. On the other hand, models 2 and 4 showed the lowest correlation value for both HALE at birth (0.674) and HALE at 65 years-old (0.786). Reliability followed the same pattern, with the highest values for the comparison between model 1 and 2, both for HALE at birth (0.986) and HALE at 65 years-old (0.988), and the lowest values for the comparisons between model 2 and 4 for HALE at birth (0.741) and HALE at 65 years-old (0.751). Disagreement was quite low for all comparisons, being the lowest values being between models 1 and 2 for both HALE at birth (0.011) and HALE at 65 years-old (0.014). Spearman’s rank correlation, reliability, and disagreement estimates among the five models, for HALE at birth and HALE at 65 years-old, are presented in Table 3. Similar results were found using education attainment as control (Supplementary Table 2).

**Table 3:** Spearman’s rho correlation, intraclass correlation coefficient (ICC) and information based measure of disagreement (IBMD) between efficiency scores of stochastic frontier models of health system efficiency using healthy life expectancy (HALE) at birth and HALE at 65 years old as outputs, in 2015 for 188 countries

	**HALE at birth**					**HALE at 65 years-old**				
	Model 1	Model 2	Model 3	Model 4	Model 5	Model 1	Model 2	Model 3	Model 4	Model 5
	Spearman’s r (95% CI)					Spearman’s r (95% CI)				
Model 1		0.981 (0.970–0.987)	0.796 (0.717–0.854)	0.722 (0.619–0.798)	0.871 (0.811–0.914)		0.986 (0.978–0.990)	0.929 (0.896–0.953)	0.814 (0.740–0.866)	0.946 (0.924–0.959)
Model 2			0.732 (0.636–0.804)	0.674 (0.570–0.758)	0.779 (0.694–0.840)			0.876 (0.828–0.911)	0.786 (0.715–0.845)	0.888 (0.850–0.916)
Model 3				0.884 (0.835–0.916)	0.858 (0.806–0.897)				0.864 (0.810–0.906)	0.970 (0.944–0.983)
Model 4					0.766 (0.677–0.828)					0.834 (0.768–0.885)
	ICC (95% CI)					ICC (95% CI)				
Model 1		0.986 (0.944–0.994)	0.852 (0.728–0.911)	0.809 (0.587–0.896)	0.931 (0.897–0.953)		0.988 (0.964–0.994)	0.852 (0.717–0.913)	0.800 (0.709–0.859)	0.890 (0.736–0.943)
Model 2			0.783 (0.566–0.877)	0.741 (0.422–0.864)	0.868 (0.769–0.918)			0.782 (0.574–0.874)	0.751 (0.606–0.836)	0.820 (0.588–0.905)
Model 3				0.942 (0.919–0.958)	0.925 (0.883–0.949)				0.887 (0.852–0.914)	0.969 (0.958–0.976)
Model 4					0.870 (0.764–0.921)					0.850 (0.804–0.885)
	IBMD (95% CI)					IBMD (95% CI)				
Model 1		0.011 (0.010–0.012)	0.027 (0.023–0.030)	0.031 (0.027–0.034)	0.020 (0.018–0.022)		0.014 (0.012–0.015)	0.030 (0.024–0.036)	0.040 (0.034–0.047)	0.031 (0.027–0.036)
Model 2			0.034 (0.030–0.038)	0.038 (0.033–0.042)	0.028 (0.026–0.033)			0.042 (0.036–0.049)	0.047 (0.041–0.055)	0.044 (0.037–0.049)
Model 3				0.014 (0.012–0.016)	0.019 (0.017–0.021)				0.030 (0.026–0.034)	0.012 (0.010–0.014)
Model 4					0.024 (0.021–0.026)					0.034 (0.029–0.039)

**Fig. 3:**
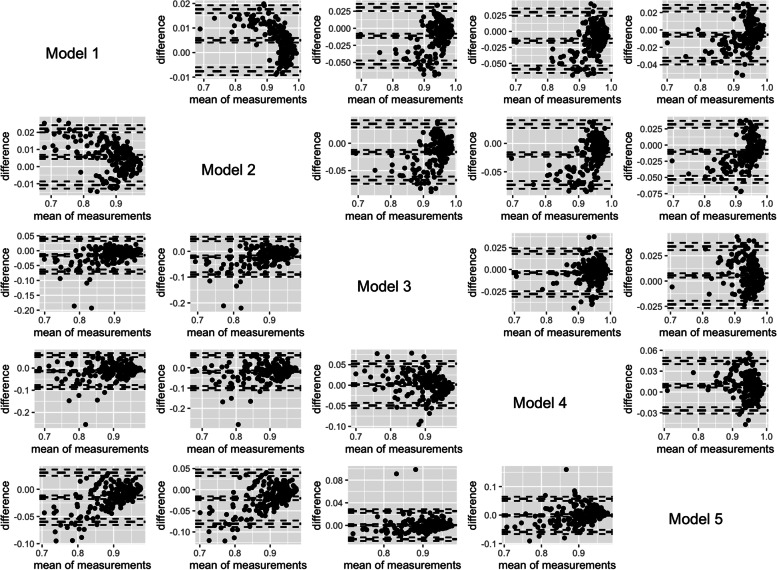
Matrix of Bland–Altman plots comparing the pairwise differences between efficiency estimates for HALE at birth (upper-diagonal) and HALE at 65 years (lower diagonal), in 2015 for 188 countries. Horizontal lines show the mean difference and the 95% CI of limits of agreement

Figure 3 shows the Bland–Altman plots for the differences between all the estimates for each input model, for HALE at birth, in the upper right corner, and for HALE at 65 years-old, in the lower left corner. Each point within the plots represents one estimate from a specific country in that specific output. While for HALE at birth there is a greater dispersion of measures for higher efficiency scores for HALE at 65 years-old, the dispersion of results occurs mainly for medium–low values.

## Discussion

We performed a worldwide cross-sectional efficiency study for 188 countries, in 2015. We present different approaches for adjusting health expenditure per capita to the age structure and assessing their influence on health systems efficiency scores, proposing a method to adjust health expenditure for age, through a standardisation process.

Assessing health systems’ efficiency poses major challenges given the difficulties of properly defining and evaluating inputs and outputs, the complexity of the relationship between them, and its dependency on factors other than health system inputs [43].

Health expectancy is seen as a comprehensive output/outcome measure, being a mortality-morbidity indicator. Health expectancy indicators account for age considering they are calculated by combining standard life tables information on mortality with age-sex-specific prevalence data for health states using Sullivan’s method [28]. In this study, we used both health life expectancy at birth and at 65 years old. Nevertheless, similar results on age-adjustement would be expected for similar population health metrics such as life expectancy or mortality.

Regarding the inputs, health expenditure is one of the most commonly considered when estimating health systems efficiency. This indicator itself can depend on several other factors, including age structure. In fact, it is important to highlight that several efforts have been made to estimate health expenditure by age group [14, 34, 44–46]. Even though proximity to death seems to be the most appropriate approach for health expenditure prediction [16, 18, 47–51], age structure might be a good proxy and summary of proximity to death and could, therefore, be included in efficiency analysis to account for the aforementioned factor. Moreover, proximity to death is pointed in the literature as a key driver of health spending despite some comentators arguing that higher per capita costs are not entirely attributable to end-of-life care. Country-level data also show an increase in the probability of using health services as age increases, along with a more frequent and intensive utilization [30].

In fact, when analysing the differences of unadjusted total health expenditure per capita and age-adjusted health expenditure per capita, the differences are huge as seen in panels E and F of Fig. 1, with implications for the results of the efficiency analyses. For some particular countries, these differences represent up to approximately 2700 US$ PPP less than the actual HEpC due to the differences in age structure. With age-adjustment, some countries halve their HEpC when using the subset of OECD subset as reference.

For the calculation of efficiency scores, we chose SFA, a parametric method that assumes that the difference from an estimated average parameter is due to both a systematic error and an inefficiency term. This implies assumptions about the expected distribution of the error term, which may be inappropriate [52]. SFA estimates a cost production function as the result of a relationship between inputs and outputs variables, but it is also important to consider environmental variables in this process for better efficiency estimates, as inputs and outputs are mainly driven by non-health care determinants that need to be controlled for [53, 54]. Environmental variables, namely age structure, allow the characterization of the population they serve, which may both influence the outcomes obtained and the result of efficiency effects.

In this study, we also propose an age-adjusted model, Model 2, which summarizes information on demographic data and health financing into a single measure, instead of adding other population age structure variables, thus being more easily adaptable for other analysis in other fields besides efficiency. In model 2, we used indirect age adjustment to integrate the influence of age structure into a single variable, by choosing a standard population that, in our case, corresponds to a subset of OECD countries [34]. The remaining models address the influence of the age variable considering other aging indicators, such as the ageing index or proportion of inhabitants aged over 65 years-old.

Model 4 presents a more granular demographic characterization of the population, as it considers age groups with a range of 5 years. On the other side, model 2 results from the indirect adjustment from available age-adjusted values for the OECD subset population health expenditure, being more sensitive to the sample composition.

Spearman’s rank correlations are conventionally used to compare efficiency estimates provided by different model specifications to make validity judgments [40]. Models 1 and 2, i.e. using unadjusted health expenditure and age-adjusted health expenditure as inputs, showed the highest correlation and reliability in models’ comparison, as well as the lowest disagreement. This does not mean that, in order to adjust for age structure, model 2 is the best but rather that it is the closest to model 1. Nevertheless, despite the overall good performance observed for correlation, reliability and disagreement between models, for HALE at 65 years-old, the Bland–Altman matrix plots suggest that the variation cannot be disregarded. This implies that the choice of method used to assume age structure for efficiency analysis may have repercussions on the analysis and interpretation of results for countries with lower efficiency scores.

Overall, there was a strong and positive relationship between efficiency scores estimated with non-adjusted and age-adjusted health expenditure. However, to compare health systems’ efficiency without age-adjustment can still be an unfair judgment and analysis. For example, Persian Gulf countries are the most impacted when comparing both age-adjusted and crude health expenditure per capita as they present one of the youngest age structures worldwide. The opposite happens with Japan.

However, the main question remains: “So what type of age-adjustment should we consider when performing a health system efficiency analysis?”. While correlation, reliability, and disagreement do not answer the question, the goodness-of-fit estimates (AIC, BIC and likelihood ratio test) suggest that using an age-adjusted health expenditure per capita (model 2) might be the best option, comparing to age groups’ proportions or ageing index.

To the best of our knowledge, this is the first study to propose and evaluate four distinct age-adjusted health expenditure per capita models in health systems efficiency analysis and to compare them with each other, as well as with the unadjusted model. Health policymakers and researchers should not be indifferent to the methods of age adjustments in frontier models used to score efficiency. In fact, as efficiency analyses are comparisons of productivities with the best performing decision making units, they should be as adequate and fair as possible. As age structure is a difficult to change variable for the health production function that can impact the efficiency scores. It is thus important to adjust for it. In order to do so, age-adjusted health expenditure per capita seems to be the best option. Additionally, if it is the chosen option and other input variables need to be considered, this might avoid further interactions between age variables and other inputs. Nevertheless, in analyses with many inputs that can be affected by age structure of the population and cannot be adjusted for it due to lack of data by age group, considering proportions of age groups can be an option, although interactions should be tested.

Given the importance of national health care systems efficiency scores and identifying factors that cause inefficiency to support the design policies to raise health care efficiency, the accuracy of efficiency quantification evidence is key. Our study demonstrates that despite producing reasonable estimations, the precision of the common approach can be improved by adopting an age-adjusted model. The implications of these results highlight the relevance of the initial step in health efficiency studies in which health expenditure data availability, trustworthiness and detail is assessed, since the choice of these inputs has the potential to improve the quantification.

Nevertheless, the scope of this work is to open the discussion on how to modulate the effect of environmental variables on health systems efficiency analysis, such as age structure. The differences found in the efficiency scores between models, particularly in the case of countries with lower efficiency scores, must be taken into account when comparing and interpreting the literature on health systems efficiency analysis.

## Limitations

One of the main limitations of this study is that the standard population selected for input adjustment, the OECD subset population, is different from the standard population chosen for output adjustment, the Global Burden Disease (GBD) standard population. Second, this study only considered age structure as the sole environmental determinant of health expenditure but, as mentioned above, health status and longevity are also factors whose effect should not be underestimated. Furthermore, as the focus of the article was to investigate the effects of different approaches for accounting health expenditures adjusted for age on health system efficiency estimates, we have not assessed the relationship between efficiency scores and other environmental determinants beyond age and education (e.g. income, behavioural risks, etc.).

## Conclusion

Age structure (or proximity to death) influences health expenditure, which in turn is usually used as input of health systems efficiency analyses. However, while population age structure is usually taken into account to adjust health system outputs for efficiency analyses, health expenditure or other inputs are not often adjusted. This study proposes a method of integrating age structure for age-adjusted health expenditure and compares it with other approaches. Although different models showed relatively good correlation, reliability and low disagreement considering the efficiency scores obtained, there is important variability when age structure is considered that cannot be disregarded. The proposed model seems to be an interesting option for this methodological question, while further studies using different data and contexts can build on our results.

## Supplementary Information

**Additional file 1.** 

**Additional file 2.**

## Data Availability

All data used in this study is publicly available in the described sources.

## Abbreviations

AIC: Akaike information criteria
BIC: Bayesian information criteria
CI: Confidence interval
HALE: Healthy life expectancy
HEpC: Health Expenditure per Capita
IBMD: Information-based measure of disagreement
ICC: Intraclass correlation coefficient
PPP: Purchasing-power parity
SFA: Stochastic frontier analysis
