# Supplementary material for: Should we adjust health expenditure for age structure on health systems efficiency? A worldwide analysis
Source: Health Econ Rev. 2023 Feb 13;13:11. doi: 10.1186/s13561-023-00421-2 (PMC9926817; doi:10.1186/s13561-023-00421-2)
Supplement: Supplementary file 1 — Additional file 1. [file 13561_2023_421_MOESM1_ESM.docx]

Supplementary Table 1

|  | HALE at birth | | | | | | | | | | HALE at 65 years-old | | | | | | | | | |
| --- | --- | --- | --- | --- | --- | --- | --- | --- | --- | --- | --- | --- | --- | --- | --- | --- | --- | --- | --- | --- |
|  | Min | Q1 | Median | Q3 | Max | Mean | SD | AIC | BIC | LR P-value | Min | Q1 | Median | Q3 | Max | Mean | SD | AIC | BIC | LR P-value |
| Model 1 | 0.675 | 0.915 | 0.946 | 0.967 | 0.991 | 0.932 | 0.049 | -518.3 | -502.2 | 9.2 e-10 | 0.679 | 0.864 | 0.909 | 0.934 | 0.977 | 0.890 | 0.065 | -275.9 | -259.8 | 1.7 e-4 |
| Model 2 | 0.665 | 0.907 | 0.942 | 0.967 | 0.991 | 0.929 | 0.052 | -507.2 | -491.1 | 1.8 e-10 | 0.660 | 0.856 | 0.903 | 0.932 | 0.977 | 0.883 | 0.071 | -261.7 | -245.6 | 0.4 e-4 |
| Model 3 | 0.695 | 0.929 | 0.953 | 0.970 | 0.992 | 0.943 | 0.040 | -562.3 | -536.5 | 19.7 e-10 | 0.746 | 0.894 | 0.927 | 0.944 | 0.974 | 0.915 | 0.043 | -305.5 | -279.7 | 498.1 e-4 |
| Model 4 | 0.706 | 0.936 | 0.957 | 0.973 | 0.994 | 0.947 | 0.038 | -561.2 | -487.1 | 1859  e-10 | 0.707 | 0.881 | 0.924 | 0.949 | 0.980 | 0.909 | 0.054 | -316.6 | -242.5 | 73.9 e-4 |
| Model 5 | 0.695 | 0.923 | 0.947 | 0.966 | 0.989 | 0.937 | 0.043 | -532.1 | -512.8 | 1491  e-10 | 0.769 | 0.900 | 0.926 | 0.942 | 0.974 | 0.917 | 0.040 | -295.2 | -275.9 | 1015 e-4 |
