# Supplementary material for: Should we adjust health expenditure for age structure on health systems efficiency? A worldwide analysis
Source: Health Econ Rev. 2023 Feb 13;13:11. doi: 10.1186/s13561-023-00421-2 (PMC9926817; doi:10.1186/s13561-023-00421-2)
Supplement: Supplementary file 2 — Additional file 2. [file 13561_2023_421_MOESM2_ESM.docx]

Supplementary Table 2

|  | **HALE at birth** | | | | | **HALE at 65 years-old** | | | | |
| --- | --- | --- | --- | --- | --- | --- | --- | --- | --- | --- |
|  | Model 1 | Model 2 | Model 3 | Model 4 | Model 5 | Model 1 | Model 2 | Model 3 | Model 4 | Model 5 |
|  | Spearman’s r (95% CI) | | | | | Spearman’s r (95% CI) | | | | |
| Model 1 |  | 0.991  (0.986-0.994) | 0.859  (0.801-0.898) | 0.772  (0.687-0.837) | 0.933  (0.901-0.953) |  | 0.991  (0.985-0.994) | 0.905  (0.857-0.937) | 0.797  (0.721-0.849) | 0.924  (0.895-0.945) |
| Model 2 |  |  | 0.834  (0.765-0.884) | 0.751  (0.664-0.813) | 0.890  (0.841-0.921) |  |  | 0.860  (0.798-0.903) | 0.769  (0.694-0.832) | 0.876  (0.832-0.908) |
| Model 3 |  |  |  | 0.889  (0.846-0.922) | 0.891  (0.841-0.920) |  |  |  | 0.859  (0.806-0.900) | 0.957  (0.926-0.976) |
| Model 4 |  |  |  |  | 0.790  (0.715-0.848) |  |  |  |  | 0.823  (0.758-0.872) |
|  | ICC (95% CI) | | | | | ICC (95% CI) | | | | |
| Model 1 |  | 0.993  (0.978-0.997) | 0.886  (0.760-0.936) | 0.822  (0.589-0.906) | 0.955  (0.929-0.971) |  | 0.987  (0.943-0.994) | 0.750  (0.397-0.874) | 0.761  (0.604-0.848) | 0.745  (0.310-0.881) |
| Model 2 |  |  | 0.844  (0.651-0.916) | 0.777  (0.480-0.885) | 0.918  (0.851-0.950) |  |  | 0.671  (0.267-0.830) | 0.704  (0.465-0.823) | 0.662  (0.204-0.833) |
| Model 3 |  |  |  | 0.940  (0.913-0.958) | 0.939  (0.898-0.961) |  |  |  | 0.864  (0.818-0.898) | 0.958  (0.944-0.968) |
| Model 4 |  |  |  |  | 0.875  (0.754-0.928) |  |  |  |  | 0.799  (0.732-0.850) |
|  | IBMD (95% CI) | | | | | IBMD (95% CI) | | | | |
| Model 1 |  | 0.008  (0.007-0.008) | 0.024  (0.021-0.027) | 0.032  (0.028-0.035) | 0.017  (0.015-0.019) |  | 0.014  (0.012-0.016) | 0.041  (0.034-0.048) | 0.045  (0.039-0.052) | 0.045  (0.038-0.051) |
| Model 2 |  |  | 0.029  (0.025-0.033) | 0.037  (0.032-0.041) | 0.023  (0.021-0.026) |  |  | 0.053  (0.044-0.060) | 0.054  (0.047-0.062) | 0.056  (0.048-0.064) |
| Model 3 |  |  |  | 0.014  (0.012-0.017) | 0.017  (0.015-0.019) |  |  |  | 0.030  (0.026-0.033) | 0.012  (0.010-0.014) |
| Model 4 |  |  |  |  | 0.024  (0.022-0.027) |  |  |  |  | 0.035  (0.030-0.039) |
